# Visible AI assistant outputs in psychosocial risk management: an OHP/OHS-grounded benchmark for governance and responsible use

**DOI:** 10.3389/fpubh.2026.1857113

**Published:** 2026-07-15

**Authors:** Mehmed Zahid Çögenli

**Affiliations:** Department of Occupational Health and Safety, Faculty of Health Sciences, Uşak University, Uşak, Türkiye

**Keywords:** AI assistants, benchmarking, governance, ISO 45003, occupational health and safety, occupational health psychology, psychosocial risk management, reporting

## Abstract

**Background:**

This study introduces and applies an occupational health psychology (OHP) and occupational health and safety (OHS)-grounded benchmark for evaluating visible artificial intelligence (AI) assistant outputs in psychosocial risk management.

**Methods:**

Five user-facing general-purpose AI assistant products were evaluated across four locked psychosocial-risk scenarios, three repeated runs, and four fixed tasks per conversation, yielding 60 conversations and 240 task-level outputs. Outputs were scored on risk identification and differentiation, multi-level organizational framing, preventive organizational actionability, and professional boundedness and verification. A blind second-rater layer and focused adjudication were used to test profile stability and resolve profile-changing disagreements.

**Results:**

Exact agreement was observed in 153 of 240 task rows (63.7%), while 239 rows (99.6%) were exact or within one point. Profile mismatches were concentrated in Gemini and Le Chat/Mistral rather than distributed across scenarios or runs. Under the final consensus structure, 36 conversations were classified as Structurally Usable Support, 23 as Mixed/Partially Usable Support, and 1 as Problematic Support.

**Conclusion:**

The main contribution is not a generic ranking of assistant products, but a domain-specific evaluation logic for psychosocial risk management, together with a reviewer-facing process model and derived governance and reporting guides for more transparent and responsible organizational use.

## Introduction

General-purpose artificial intelligence (AI) assistants are becoming increasingly visible in workplace information, drafting, reflection, and judgment-support tasks. In organizational settings, the relevant question is no longer only whether these systems can generate fluent responses, but how their visible outputs should be evaluated when they may shape how workplace issues are framed, prioritized, and acted upon. This matters especially in a policy environment where workplace AI is increasingly approached not only as a productivity tool, but also as a source of governance questions concerning accountability, oversight, work quality, inequality, and the responsible use of automated decision support (1, 11). Organisation for Economic Co-operation and Development (OECD) analyses capture this duality clearly by treating workplace AI as a source of both potential gains and material organizational risks (1).

This issue becomes especially consequential in psychosocial risk management. In occupational health psychology and occupational health and safety, psychosocial risks are not treated as merely individual distress, private coping difficulty, or generic wellbeing concerns. Rather, they are located in the design, organization, and management of work, as well as in the wider social context in which work is carried out. The European Agency for Safety and Health at Work (EU-OSHA) defines psychosocial risks in relation to poor work design, organization and management, and adverse social context at work, while the International Labour Organization (ILO) similarly states that anything in the design or management of work that increases the risk of work-related stress can be understood as a psychosocial hazard (2, 3). International Organization for Standardization (ISO) 45003 reinforces this orientation by placing psychosocial risk management within an occupational health and safety management system based on ISO 45001 and by explicitly framing the guidance as applicable across organizations of all sizes and sectors (4). Taken together, these sources position psychosocial risk management as an organizational and prevention-oriented domain rather than a generic advice domain. This position is also consistent with recent synthesis evidence showing that several working conditions are associated with increased risk of mental health problems and that workplace improvement depends on organizational and preventive intervention rather than individual-level framing alone (5).

That framing has direct implications for how AI outputs should be evaluated in this domain. If AI assistants are used in psychosocial-risk-related reflection, preliminary assessment, drafting, or documentation support, the relevant question is not whether they simply sound polished or plausible. The more important question is whether they can identify psychosocial risks without reducing organizational problems to the individual level, preserve prevention-oriented reasoning, maintain multi-level organizational framing, and remain appropriately bounded for professional use. This emphasis is consistent with the World Health Organization (WHO) Guidelines on Mental Health at Work, which recommend organizational interventions alongside manager training and worker training, and with the WHO/ILO policy brief, which frames the prevention of psychosocial risks, the protection and promotion of mental health at work, and the support of workers with mental health conditions as actionable responsibilities for governments, employers, workers, and their organizations (6, 7). Under this logic, a fluent answer may still be professionally weak if it individualizes risk, blurs the distinction between hazards and consequences, neglects preventive organizational action, or fails to state verification boundaries.

This also exposes a limitation in the broader language-model evaluation literature. Benchmarking efforts such as Holistic Evaluation of Language Models (HELM) have significantly advanced transparency by organizing evaluation around scenarios and multiple desiderata rather than around a single score, while large-scale survey work on large language model (LLM) evaluation has mapped the rapidly expanding ecosystem of what to evaluate, where to evaluate, and how to evaluate across diverse tasks and domains (8, 9). That literature is highly valuable, but it does not by itself resolve how visible AI assistant outputs should be judged in psychosocial risk management, where the central issue is not general capability alone but whether outputs remain professionally usable within a prevention-sensitive occupational context. The gap, then, is not simply a lack of more benchmarking, but a lack of benchmarking logic explicitly grounded in occupational health psychology and occupational health and safety principles for psychosocial risk management tasks. This need for domain-specific evaluation is also consistent with recent expert-review approaches in clinical LLM assessment, which show that task-specific, blinded human evaluation can capture performance features that are not adequately reflected by generic benchmark logic alone (10).

The present study addresses this gap by introducing and applying an occupational health psychology (OHP) and occupational health and safety (OHS)-grounded benchmark for visible artificial intelligence (AI) assistant outputs in psychosocial risk management. It examines whether outputs generated under a locked protocol remain professionally usable across four task demands: psychosocial risk identification, organizational framing, prevention-oriented action, and bounded professional use. Its contribution is not a consumer-style product ranking, but a reviewer-auditable evaluation architecture for identifying recurrent visible-output support patterns in a high-stakes occupational domain.

## Materials and methods

### Study design and analytic object

This study used a structured, reviewer-auditable benchmark to evaluate the visible outputs of leading general-purpose AI assistant products in standardized psychosocial risk management scenarios. The final benchmark archive comprised 60 conversations and 240 task-level outputs, generated through a fixed design of five assistant products, four locked scenarios, three repeated runs, and four fixed tasks per conversation. The analytic object was not abstract model capability or inferred internal reasoning, but the visible text output that an organizational user could directly encounter, archive, compare, and evaluate in practice.

The study was grounded in occupational health psychology and occupational health and safety principles and was designed specifically for psychosocial risk management rather than for generic text-quality comparison. This section defines the analytic object, protocol structure, rating process, and reliability strategy used to evaluate visible outputs under fixed benchmark conditions.

### Visible-output evaluation process model

The benchmark was organized through a visible-output evaluation process model that linked a structured sequence of benchmark stages: fixed professional context, locked scenarios and four-task sequence, standardized execution and run logging, visible-output archiving, task-level rating, and conversation-level profile assignment with governance and reporting relevance. The function of this process model was methodological rather than theoretical. It was not intended as a substantive model of worker experience or AI cognition, but as an explicit benchmark architecture showing how evaluative judgments were generated from standardized visible outputs.

**Figure 1:**
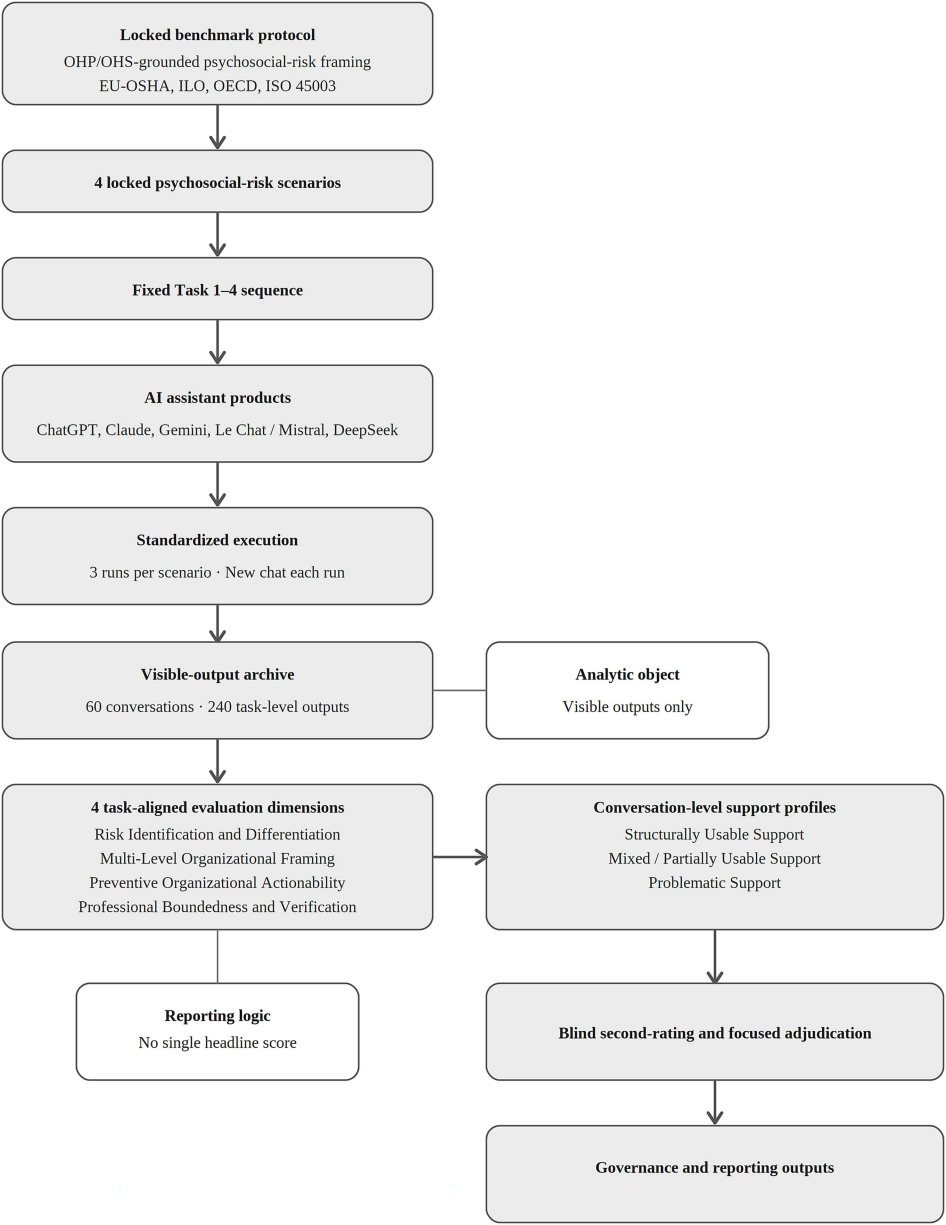
Visible-output evaluation process model. The figure summarizes the benchmark architecture from the locked protocol and standardized execution to visible-output archiving, task-aligned evaluation, support-profile assignment, blind second-rating, focused adjudication, and governance/reporting outputs.

Two additional boundary rules governed the process. First, no inference was made about hidden reasoning, backend cognition, or latent model processes. Second, visible instability in language, script, or formatting was logged separately from substantive judgment rather than being automatically folded into core task scoring. This distinction was important because an output could be substantively relevant yet still display visible instability that affected interpretability or professional trust. The overall benchmark architecture is summarized in Figure 1.

### Product set and observed benchmark environment

The benchmark included five leading general-purpose AI assistant products: ChatGPT, Claude, Gemini, Le Chat/Mistral, and DeepSeek. The comparative object was the user-facing assistant product as encountered in practice rather than an abstract base model in isolation. Product selection was deliberately bounded to user-facing, general-purpose chat assistants that could be run under the same locked multi-turn protocol during the benchmark period. The aim was not exhaustive market coverage, but bounded cross-product comparability under a fixed visible-output design.

For each run, the visible product or model label was recorded verbatim, together with any visible plan, access, thinking, search, or tool indicator shown in the interface at runtime. Where backend parameters were not transparently exposed, they were not inferred. The output constraint required English-only responding and explicitly prohibited other languages, scripts, or translated equivalent terms. Visible runtime labels were treated as observed metadata rather than as evidence about hidden reasoning processes. This choice preserved the study’s visible-output logic and kept the benchmark anchored to what a practitioner could actually see and document. Benchmark data were collected between 19 and 25 March 2026, and visible runtime labels were recorded exactly as displayed at the time of each run. The observed product environment and visible runtime labels are summarized in Table 1.

**Table 1:** Observed product environment and visible runtime labels.

Product	Visible product/model label	Visible plan/access label	Visible tool or mode label	Observed benchmark logic
ChatGPT	5.2	Free Plan	—	User-facing ChatGPT environment logged as displayed at run time.
Claude	Sonnet 4.6	Extended	Extended Thinking	Visible extended-mode label recorded verbatim from the product interface.
DeepSeek	V3	Deep Think	Deep Think	Visible deep-think label recorded as user-facing runtime metadata.
Gemini	3	Thinking	Thinking	Visible thinking label recorded as user-facing runtime metadata.
Le Chat/Mistral	LeChat	Think	Think	Visible think label recorded as user-facing runtime metadata.

### Standardized execution and fixed professional context

Execution was standardized across products and runs. A new chat was opened for each product × scenario × repetition. English was used as the operational language whenever the product exposed that option. The first prompt combined the output constraint, the fixed professional context, the full scenario block, and Task 1. Tasks 2, 3, and 4 were then delivered in the same locked order without rephrasing the case. Regeneration was not used during benchmark runs, and assistant clarification questions were not answered; such events were logged instead. The three-run structure was determined *a priori* as a bounded repeated-output design. It was intended to capture whether visible support profiles remained stable or varied across repeated user-facing executions, while keeping the corpus feasible for complete task-level scoring and blind review. With five products, four scenarios, three runs, and four tasks per conversation, the design yielded 240 task-level outputs. Temperature or sampling parameters were not set because the benchmark evaluated user-facing assistant products rather than API-controlled base models, and these parameters were not transparently exposed in the observed product interfaces. Consistent with the visible-output logic of the study, unobserved backend settings were therefore not inferred.

The fixed professional context stated that the assistant was supporting an occupational health and safety specialist with 5 years of experience in a medium-sized enterprise. The task frame was psychosocial risk pre-assessment and the structuring of an organizationally oriented intervention logic. The prompt also made explicit that the assistant’s response did not replace professional judgment, formal workplace assessment, legal review, or organizational decision authority. This framing was designed to keep the benchmark aligned with OHP/OHS practice and with prevention-oriented psychosocial risk management rather than generic workplace advice.

### Scenario architecture

The benchmark used four locked psychosocial risk scenarios organized around internationally stable OHP/OHS domains: work design and task control; social and managerial relations; workload, pace, and recovery; and organizational change, insecurity, and communication uncertainty. This four-domain structure was used to anchor the benchmark in a reproducible psychosocial risk architecture rather than in an *ad hoc* set of cases. The overall structure was consistent with major institutional treatments of psychosocial risk, which locate such risks in the design, organization, and management of work and in the broader social context of work rather than in individual weakness alone (2–4, 6).

Each scenario was designed to test a distinct but related evaluative challenge. The first scenario examined whether assistants could distinguish psychosocial risks from their consequences while preserving organizational framing. The second examined whether assistants could recognize managerial and interpersonal harm without drifting into etiquette-only or blame-centered framing. The third examined whether outputs prioritized staffing, scheduling, recovery, and work-design interventions over generic coping advice. The fourth examined whether organizational change and uncertainty were framed as governance and prevention problems requiring communication, participation, and role clarity. Full locked scenario texts and the exact Task 1–4 wording are reproduced in Supplementary File 1.

### Core task sequence

All conversations followed the same fixed four-task sequence. Task 1 asked the assistant to identify the main psychosocial risks in the scenario and distinguish those risks from their likely consequences. Task 2 required multi-level framing across individual, managerial, and organizational levels, while avoiding reduction of the problem to the individual level alone. Task 3 asked for practical prevention and intervention actions from an occupational health and safety perspective, with priority given to organizational and preventive measures rather than downstream coping-only suggestions. Task 4 required the assistant to state the limits of the AI-based assessment and clarify what would need to be verified before any professional conclusion or workplace action could be taken.

This task sequence was intentionally cumulative. It did not test isolated fragments of output quality, but whether the assistant could move from risk recognition to organizational framing, then to prevention-oriented action, and finally to bounded professional closure. In this sense, the benchmark assessed not only topical relevance, but also whether the visible output followed a professionally usable sequence for psychosocial risk management. The four evaluation dimensions were task-aligned rather than crossed with every task: Task 1 was evaluated for Risk Identification and Differentiation, Task 2 for Multi-Level Organizational Framing, Task 3 for Preventive Organizational Actionability, and Task 4 for Professional Boundedness and Verification.

### Task-level evaluation rubric and critical note structure

Task-level outputs were evaluated using a three-point ordinal rubric applied across four task-aligned dimensions: Risk Identification and Differentiation, Multi-Level Organizational Framing, Preventive Organizational Actionability, and Professional Boundedness and Verification. The rubric was designed to remain close to the locked task prompts rather than to impose a generic quality grid after the fact. In this way, scoring remained anchored to the practical judgment demands that the benchmark was intended to test.

Risk Identification and Differentiation assessed whether the assistant accurately identified the central psychosocial risks in the case and clearly distinguished those risks from their likely consequences. Multi-Level Organizational Framing assessed whether the response represented the problem across individual, managerial, and organizational levels without collapsing the issue into individual resilience, coping, or communication style alone. Preventive Organizational Actionability assessed whether the assistant translated the scenario into plausible organizational and preventive interventions rather than relying primarily on downstream coping advice. Professional Boundedness and Verification assessed whether the response remained within the case, avoided unsupported additions, stated the limits of AI-based assessment, and clarified what would need to be verified before any professional conclusion or workplace action could be justified.

Each dimension was scored on the same three-point scale. A score of 1 indicated Problematic performance, meaning that the output failed the relevant benchmark expectation in a material way. A score of 2 indicated Mixed/Partial performance, meaning that the response captured part of the required logic but remained incomplete, unstable, or only partly usable. A score of 3 indicated Structurally Usable performance, meaning that the output met the benchmark criterion in a sufficiently clear, professionally relevant, and bounded way to function as usable support within the fixed benchmark context. A score of 3 was reserved for outputs that satisfied the core task demand without material omission, whereas a score of 2 was used when the relevant logic was present but remained incomplete, uneven, or only partly defensible for professional use.

In addition to the substantive scores, each task row included a short critical note field. This field was not part of the numerical score and was used only to capture recurrent issues that were analytically important but should not automatically distort the substantive judgment. The final critical-note categories were none, individualizing drift, unsupported/fabricated addition, and visible instability. Visible instability referred to mixed-script anomalies, unexpected language switching, or formatting breakdowns that materially affected interpretability. Keeping such events separate from substantive scoring preserved the distinction between content weakness and delivery instability.

The rubric should therefore be read as a structured benchmark judgment tool rather than as a formally validated measurement instrument. It was used to support transparent, task-aligned comparison of visible outputs under fixed benchmark conditions, not to establish a psychometric scale or a latent construct measure.

### Primary scoring procedure

Primary scoring was conducted at the task-output level. Each of the 240 task rows was read in relation to the fixed professional context, the locked scenario text, and the specific task prompt to which the output responded. Scores were assigned dimension by dimension rather than through a single global impression. This approach was intended to preserve analytic separation between risk recognition, organizational framing, preventive actionability, and bounded verification.

The benchmark did not produce a single headline total score for each assistant product. Nor was it designed to identify a generic best assistant. Instead, task-level scoring served as the basis for identifying repeated response patterns, weak points, and support conditions across the benchmark archive. This design choice was consistent with the study’s broader aim of evaluating visible AI outputs in psychosocial risk management as a domain-specific organizational-use problem rather than as a consumer-style product contest.

### Conversation-level profile assignment

Task-level ratings were subsequently translated into conversation-level support profiles. Each conversation was assigned to one of three typology categories: Structurally Usable Support, Mixed/Partially Usable Support, or Problematic Support. The purpose of this step was not to collapse the benchmark into a single product score, but to summarize whether the conversation as a whole crossed the threshold from merely relevant output to professionally usable support within the psychosocial risk management context. Profile assignment followed a rule-guided holistic judgment: conversations were classified by reading the four task-aligned outputs together against the fixed benchmark logic, with particular attention to whether any weakness represented a local incompleteness or a broader breakdown in risk differentiation, organizational framing, preventive actionability, or bounded verification.

A conversation was considered Structurally Usable Support when its outputs consistently maintained risk differentiation, organizational framing, prevention-oriented actionability, and bounded verification without material breakdown in the task sequence. A conversation was considered Mixed/Partially Usable Support when relevant material was present but remained uneven, incomplete, or only partly defensible across tasks. A conversation was considered Problematic Support when the output failed to sustain the minimum structure required for professionally usable support and instead showed serious drift, flattening, or inadequacy across the benchmark logic. This conversation-level typology was the main interpretive layer used for final pattern reading.

### Blind second-rating and reliability strategy

After primary scoring, a blind second-rater layer was applied to test whether profile interpretations remained stable under independent re-evaluation. The blind second rater was an independent reviewer with advanced expertise in occupational health and safety: an academic with a PhD in OHS, an A-class occupational safety specialist, and a university OHS coordinator. Blind review was conducted on archived visible outputs and locked task/scenario materials without access to the primary rater’s scores, notes, or profile assignments. The role of this layer was not to mechanically average scores or to convert the benchmark into a conventional inter-rater product ranking exercise. Instead, it served two narrower purposes: first, to examine the degree and structure of agreement on the fixed rubric; and second, to identify whether any disagreements materially changed conversation-level profile assignment. In this design, “blind” refers specifically to the second rater’s lack of access to the primary scores, critical notes, and conversation-level profile assignments; it does not imply blinding to the visible product outputs themselves.

Reliability was therefore treated as an evaluation-design check rather than as psychometric validation of a latent scale. Exact agreement, within-one agreement, mean absolute difference, weighted kappa, and disagreement concentration were interpreted together. This approach was chosen because the benchmark used an ordinal three-band rubric for applied comparative judgment, not an instrument intended to maximize fine-grained score interchangeability.

### Focused adjudication

Where blind disagreement changed the conversation-level profile, those conversations were retained for focused adjudication. Adjudication was therefore targeted rather than global. The benchmark was not fully rescored from scratch after the second-rater step; instead, only conversations with profile-changing disagreement were included, and within those conversations only task rows with actual scoring disagreement were re-examined for final adjudication. Focused adjudication was conducted by the primary author after completion of the blind second-rating stage. The primary and blind ratings and notes were compared, but the full archive was not rescored. No majority-rule procedure, automated averaging, expert-panel vote, or third-party arbitration was used. Instead, adjudication was limited to the 24 conversations in which blind review changed the conversation-level profile, and within those conversations only the 61 task rows with actual score disagreement were reconsidered. Each disputed row was re-examined against the archived visible output, the locked scenario, the exact task prompt, the relevant rubric anchor, the recorded critical-note context, and the practical consequence for the final conversation-level support profile.

Consensus decisions were based on the archived visible output, the locked scenario and task prompt, the fixed rubric anchor, the recorded critical-note context, and the practical consequence for conversation-level profile assignment. In other words, the unit of adjudication was not an isolated numerical discrepancy alone, but the extent to which a disputed row altered the final support interpretation of the conversation. This kept adjudication reviewer-auditable while preventing indiscriminate rescoring of the full archive.

### Statistical and descriptive reliability reporting

Inter-rater agreement was summarized using exact agreement counts, within-one agreement, mean absolute difference, and weighted kappa estimates appropriate for ordinal judgments. To make the reliability pattern interpretable from more than one angle, both linear weighted kappa and quadratic weighted kappa were calculated, alongside Spearman’s rho as a descriptive rank-order indicator. Conversation-level profile agreement was also reported descriptively.

Given the benchmark’s ordinal structure and its emphasis on profile stability rather than on a single aggregate score, reliability was interpreted as evidence about the consistency and concentration of evaluative judgments rather than as a psychometric validation exercise. The purpose of the reliability layer was narrower and more practical: to test whether the fixed rubric generated interpretable agreement, to reveal where disagreement concentrated, and to support a defensible focused adjudication process when profile-level differences emerged.

For the weighted kappa estimates, 95% confidence intervals were estimated using non-parametric cluster-bootstrap resampling at the conversation level. This approach was used because task-level rows were nested within conversations rather than treated as fully independent observations. Bootstrap samples were drawn by resampling conversations with replacement and retaining their associated task rows, while preserving the fixed three-category ordinal rating structure.

### Governance, reporting, and process-model relevance

The benchmark also generated a derived contribution layer beyond task scoring and conversation profiles. Because the evaluation design recorded fixed context, locked task structure, visible runtime metadata, critical-note issues, blind review, and adjudication structure, it supported three linked downstream outputs: a reviewer-facing process model, a governance-oriented guide, and a benchmark reporting guide. These were not treated as separate empirical datasets, but as derived outputs anchored in the benchmark architecture and the recurrent result patterns.

For this reason, the study was organized through a visible-output evaluation process model that linked protocol lock, standardized execution, visible-output archiving, task-level scoring, conversation-level profiling, focused consensus logic, and downstream governance and reporting outputs. The process model was methodological rather than substantive. It was introduced to make the evaluation architecture explicit and reviewer-facing, not to propose a theory of worker behavior or a conceptual model of AI cognition.

### Scope and analytic boundaries

Several analytic boundaries were fixed in advance. First, the study evaluated visible outputs only and did not infer hidden internal reasoning, latent model cognition, or backend decision processes. Second, product labels, plan/access labels, and tool or thinking indicators were treated as visible runtime metadata rather than as evidence about hidden mechanisms. Third, output clarity or stylistic neatness was not scored as a substantive benchmark dimension; such issues were captured only descriptively when needed. Fourth, the benchmark was confined to a fixed OHP/OHS professional context, four locked scenarios, and a locked four-task sequence, and therefore should not be read as exhausting all possible workplace uses of AI assistants.

These boundaries define the proper scope of the study. The benchmark was designed to test whether visible assistant outputs can be evaluated in a structured and professionally meaningful way under standardized psychosocial risk management conditions. That is the level at which its claims are intended to hold.

## Results

### Corpus and reporting frame

The final benchmark corpus comprised 60 conversations and 240 task-level outputs, generated through a fixed design of five assistant products, four locked psychosocial-risk scenarios, three repeated runs, and four fixed tasks per conversation. All 60 conversations were retained in the cleaned analysis package. In line with the visible-output logic of the study, the results were not collapsed into a single headline product score. Instead, the benchmark was read through task-level judgments, conversation-level support profiles, inter-rater agreement structure, and the adjudication-sensitive patterning that emerged where profile assignments diverged.

### Inter-rater agreement and reliability pattern

Across the 240 scored task rows, exact agreement was observed in 153 cases (63.7%), while 239 cases (99.6%) were either exact or within one point. The mean absolute difference was 0.367, the linear weighted Cohen’s kappa was 0.364 (95% cluster-bootstrap confidence interval [CI]: 0.265–0.474), the quadratic weighted kappa was 0.369 (95% cluster-bootstrap CI: 0.271–0.479), and Spearman’s rho was 0.477. Only one 2-point disagreement (3 vs. 1) was observed across the full benchmark, in Task 1 Risk Identification and Differentiation for one Le Chat/Mistral conversation. On exact-category terms, the reliability pattern was modest, but disagreement remained overwhelmingly concentrated in adjacent-category shifts rather than in direct reversals between clearly problematic and clearly usable judgments. The practical implication of this pattern is not that the two raters were fully interchangeable at the exact-category level. Rather, the reliability layer identified where applied judgment boundaries were sensitive. The high within-one agreement, the rarity of cross-band reversals, and the concentration of profile-changing disagreement in a limited subset of product families supported the use of focused adjudication rather than mechanical score averaging or a single aggregate ranking.

Taken together, these statistics indicate modest exact-category agreement combined with high within-one agreement, which should be interpreted cautiously in a three-category ordinal rubric because only 1–3 disagreements fall outside that band. The close proximity of the linear and quadratic weighted kappa estimates suggests that most disagreements remained adjacent-category shifts rather than heavily penalized cross-band reversals. In this applied benchmark, the second-rater layer therefore functioned as an independent reliability check and boundary-sensitivity test rather than as a basis for mechanical score averaging or as validation of a latent measurement scale. Spearman’s rho showed a moderate rank-order association, consistent with the broader pattern of directional alignment without exact-category interchangeability.

### Profile-level disagreement was localized rather than diffuse

At the conversation level, 36 conversations yielded matching support profiles, while 24 generated profile mismatches and therefore required adjudication. Here, ‘36 matches’ refers to pre-adjudication profile agreement between raters, not to the final number of Structurally Usable conversations. Prior to adjudication, all 24 mismatches were confined to two product families: Gemini and Le Chat/Mistral, with 12 mismatches each. Claude, ChatGPT, and DeepSeek each showed 12 out of 12 profile matches. Within that product-family concentration, mismatches were evenly distributed across the four scenarios, with 6 mismatches per scenario, and across the three runs, with 8 mismatches per run.

The pattern therefore pointed to profile-family concentration rather than scenario-driven or run-driven instability. In other words, disagreement was not spread diffusely across the benchmark as a whole; it was localized within a limited subset of repeated output families.

### Focused adjudication clarified repeated output-profile patterns

Because profile-changing disagreement was confined to a limited subset of cases, adjudication was conducted in a targeted rather than global manner. All 24 profile-mismatch conversations were retained for review, but only the task rows with actual scoring disagreement were re-examined for consensus. This reduced the adjudication set from 96 task rows within the mismatch conversations to 61 disputed task rows. Of the 96 task rows contained in the 24 profile-mismatch conversations, 61 showed actual score disagreement and were retained for adjudication, whereas the remaining 35 were excluded because both raters had assigned the same score. Adjudication therefore functioned as a concentrated review of the rows that actually drove profile divergence rather than as a rescore of the wider archive.

The disputed rows were not randomly distributed across the rubric. Because final adjudication was conducted after comparison of the primary and blind ratings and notes, the profile decisions reported below should be read as final adjudicated profile decisions rather than as mechanically averaged scores or majority-rule outcomes. Gemini disagreement was concentrated mainly in Risk Identification and Differentiation and Professional Boundedness and Verification. Le Chat/Mistral disagreement was concentrated in Risk Identification and Differentiation, Preventive Organizational Actionability, and Professional Boundedness and Verification, with one additional disputed row in Multi-Level Organizational Framing. The adjudication burden was therefore concentrated in repeated dimensions rather than distributed uniformly across the benchmark.

The consensus notes also clarified how these profile decisions were made. In the Gemini family, repeated downgrades were typically triggered by stricter threshold judgments on Task 1 and Task 4 despite preserved risk-consequence separation and explicit verification language across the conversation sequence; these conversations were therefore retained in the Structurally Usable Support band. In Le Chat/Mistral, repeated disagreements more often involved compressed or table-led outputs with thinner preventive and boundedness reasoning across tasks; these were generally resolved into the Mixed/Partially Usable Support band. The single Problematic case, CV-S03-MISTRAL-R2, remained low-end because it contained the only 2-point disagreement in the corpus and did not recover sufficient analytical depth in later tasks.

### Final consensus profile pattern

Under the final consensus structure adopted for manuscript closure, the benchmark preserved the main profile distinction revealed by the blind review without reducing the study to a winner-based comparison. Gemini outputs were retained in the Structurally Usable Support band. Most Le Chat/Mistral outputs were resolved into the Mixed/Partially Usable Support band, and one Le Chat/Mistral conversation remained a low-end outlier in the Problematic Support category. Closed profile matches from the non-adjudicated set remained unchanged, which meant that all ChatGPT and Claude conversations remained in Structurally Usable Support and all DeepSeek conversations remained in Mixed/Partially Usable Support.

Under this final consensus structure, the benchmark yielded 36 conversations classified as Structurally Usable Support, 23 as Mixed/Partially Usable Support, and 1 as Problematic Support. Table 2 shows the final conversation-profile distribution by product. The main empirical result was therefore a recurrent support-profile pattern across visible outputs rather than a single aggregate ordering of assistant products. These profile distributions should be read as observed patterns within the specific product environments documented during the 19–25 March collection window, not as fixed claims about the underlying products across all settings or later versions.

**Table 2:** Final consensus conversation-profile distribution by product.

Product	Structurally usable support	Mixed/partially usable support	Problematic support
ChatGPT	12	0	0
Claude	12	0	0
Gemini	12	0	0
DeepSeek	0	12	0
Le Chat/Mistral	0	11	1
Total	36	23	1

### Results summary

Taken together, the results show a stable overall benchmark structure with localized and patterned boundary sensitivity. The blind second-rater layer did not generate diffuse disagreement across scenarios or runs. Instead, disagreement remained largely adjacent-category, clustered in a limited subset of conversations, and concentrated within repeated output-profile families. Box 1 summarizes the support profile typology used in the final reading.

Box 1 Support profile typology used in the final reading.

**Table:** 

Profile	Interpretive guide
Structurally usable support	The conversation provides a professionally meaningful starting point for psychosocial risk management. It identifies central psychosocial risks, distinguishes risks from likely consequences, preserves organizational and managerial framing, offers prevention-oriented organizational actions, and remains appropriately bounded by stating limits and verification needs.
Mixed/partially usable support	The conversation contains useful material but one or more core elements remain weak, incomplete, or inconsistent. Typical cases include shallow organizational framing, procedural but thin action logic, or only partial handling of limits and verification.
Problematic support	The conversation materially fails the benchmark logic. Typical cases include major individualization drift, weak or absent organizational causation, reliance on generic wellness advice in place of prevention-oriented intervention, unsupported additions, or non-credible professional boundaries.

Optional short qualifiers may be added when a recurrent modifier is especially salient: with individualizing drift, with verification weakness, or with visible instability. These qualifiers are analytic descriptors, not separate scoring categories.

## Discussion

### Principal interpretation

This study should not be read as a generic comparison of which AI assistant writes better. Its contribution lies in relocating AI evaluation into psychosocial risk management and asking a narrower occupational question: whether visible AI assistant outputs can provide professionally usable support under standardized OHP/OHS conditions. Within that frame, the benchmark produced a stable and interpretable structure. The blind second-rater layer did not generate diffuse disagreement across the corpus. Instead, disagreement remained overwhelmingly within one scoring band, was localized at the profile level, and concentrated in a limited subset of repeated output families.

The main empirical result is therefore not a single aggregate ordering of assistant products. It is the emergence of recurrent support profiles across visible outputs. Under the final consensus structure, most conversations fell into either Structurally Usable Support or Mixed/Partially Usable Support, while clearly Problematic Support remained rare. This pattern shows that domain-specific benchmarking can yield interpretable distinctions without collapsing all judgments into a single headline score. It also supports the decision to treat adjudication as a targeted analytic step rather than as a global rescoring exercise.

A second interpretive point concerns the separation of substantive judgment from visible anomaly. The rebuilt benchmark preserved visible instability as a separate critical-note layer rather than folding it automatically into substantive score assignment. That distinction is methodologically important. In psychosocial risk management, an output may be substantively relevant yet still be unstable in script, formatting, or delivery; conversely, a neat response may still individualize risk, flatten organizational causation, or provide weak verification logic. Treating these as the same problem would blur a central analytic distinction in applied evaluation. Output language/script consistency and visible delivery quality were treated as usability-relevant descriptive features rather than as core substantive rubric dimensions; when they materially affected interpretability, they were logged as visible instability rather than converted directly into lower substantive scores.

The DeepSeek pattern merits separate note. Unlike Gemini and Le Chat/Mistral, it did not generate profile-changing disagreement, yet all 12 conversations remained in the Mixed/Partially Usable Support band. This suggests a stable partial-support profile rather than instability: outputs remained relevant across the benchmark, but repeatedly fell short of the threshold for Structurally Usable Support.

### Contribution to OHP/OHS-grounded AI evaluation

The study makes a methodological contribution by shifting AI evaluation away from generic fluency and general capability framing toward psychosocial-risk-specific criteria. That shift is not cosmetic. In OHP/OHS, psychosocial risks are defined in relation to work design, work organization, management, and social context, rather than as private distress or coping deficits alone. EU-OSHA frames psychosocial risks in terms of poor work design, organization, management, and social context; the ILO similarly treats work-related stress and psychosocial hazards as occupational issues arising from how work is designed and managed; ISO 45003 places psychosocial risk management within an occupational health and safety management system based on ISO 45001; and WHO guidance emphasizes organizational interventions that address psychosocial risk factors rather than relying only on downstream individual support.

The benchmark extends this literature by translating general language-model evaluation concerns into domain-specific judgment criteria for psychosocial risk management. In this use case, outputs were not judged only for topical plausibility or fluent workplace language, but for whether they sustained the occupational logic required across the fixed task sequence. This positions the study as a domain-specific extension of broader large language model evaluation work rather than as an alternative to general benchmarking frameworks.

### Governance, responsible organizational use, and reporting relevance

The governance relevance of the findings follows from the study’s occupational frame and result pattern. OECD work on workplace AI emphasizes both potential gains and governance risks, while ISO 45003 treats psychosocial risk management as part of an organizational occupational health and safety management system rather than as informal individualized support. Read together, these perspectives imply that AI assistant use in psychosocial-risk-related tasks should be evaluated as a matter of responsible organizational use, not only as a convenience or productivity tool.

The guides presented in Boxes 2, 3 translate the benchmark architecture and recurrent support patterns into practical governance and reporting considerations. They are not separate empirical datasets or free-standing prescriptions; rather, they summarize how fixed prompting, visible-output archiving, task-aligned scoring, critical-note separation, blind review, adjudication, and re-evaluation cadence can support more transparent use of AI assistants in occupational-health-relevant work.

Box 2 Governance guide for responsible organizational use.

**Table:** 

Guide point	Practical meaning
Preserve organizational causation	Do not treat the worker as the main source of the problem when the case points to work design, management, workload, communication, or role conditions.
Watch for individualization drift	Be cautious when the output shifts the center of explanation toward resilience, coping, attitude, or etiquette while minimizing managerial or structural contributors.
Maintain prevention-first logic	Prefer organizational and preventive action over downstream self-help or generic wellness advice.
Require professional boundedness	Do not accept overconfident legal, policy, or causal claims that exceed the information in the case.
Require explicit verification needs	Before any professional conclusion or workplace action, the output should state what still needs to be checked or confirmed.
Log delivery instability separately	Language, script, or formatting instability may not equal substantive failure, but it can still create downstream trust and usability risks.
Keep AI clearly subordinate	Even the strongest outputs in this benchmark indicate bounded support, not autonomous decision authority.

Box 3 Benchmark reporting guide for domain-specific AI evaluation.

**Table:** 

Reporting element	What to report
Visible product label	Report the user-facing product or model label exactly as encountered.
Run context	Record visible plan/access labels, tool indicators, and other visible runtime metadata rather than inferring hidden processes.
Locked protocol	State whether the scenario set, task sequence, and execution rules were fixed across products and runs.
Dimension-level scoring	Report benchmark dimensions separately rather than collapsing them into a single prestige score.
Score-flag separation	Keep pattern flags such as individualizing drift, unsupported additions, verification weakness, or visible instability separate from the main score.
Visible instability layer	Report script anomalies, unexpected language switching, or formatting breakdowns as a distinct descriptive layer.
Blind review and adjudication	Describe the second-rater layer separately and report adjudication only where profile-changing disagreement exists.
Conversation-level profiles	Show how task-level outputs combine into overall support profiles rather than reporting isolated rows only.
Non-ranking discipline	Foreground response patterns, support conditions, and consensus typology rather than a single winner-based ordering.
Re-evaluation cadence	State whether and when the benchmark should be repeated or reviewed. For user-facing AI assistant products, benchmark-based organizational decisions or reporting should be reviewed at least annually, or semi-annually where assistants are used frequently for occupational-health-relevant support tasks or where major model, interface, access-mode, or visible product-label changes occur.

### Strengths and limitations

The study has several strengths. It evaluates visible outputs rather than inferred hidden reasoning; it uses a fixed professional context, fixed scenario architecture, and locked task sequence; it preserves analytically separate task dimensions; it includes an independent blind second-rater layer; and it uses targeted adjudication rather than uncontrolled rescoring. Together, these features make the benchmark reviewer-auditable and methodologically transparent.

At the same time, the study has clear limits. First, it evaluates user-facing assistant products under one fixed benchmark environment and therefore does not capture all possible workplace uses, product configurations, or access conditions. Second, it evaluates visible outputs only and makes no claims about hidden reasoning or backend model processes. Third, the benchmark is anchored in a psychosocial-risk-management use case and should not be read as a general evaluation of all occupational-AI applications. Fourth, the profile typology is deliberately pragmatic rather than psychometric; it is intended to support disciplined comparative judgment, not latent-scale validation.

This distinction also applies to the assessment rubric. The study demonstrates the usefulness, transparency, and reviewer-auditability of a domain-specific benchmark framework for comparing visible outputs under fixed conditions; it does not establish the rubric as a formally validated measurement instrument. For this reason, the reliability layer was interpreted through agreement, boundary sensitivity, profile stability, and focused adjudication, rather than through internal-consistency coefficients, factor-analytic claims, or a single collapsed total-score validation.

The transferability of the findings should therefore be understood in relation to this fixed benchmark architecture. The results are most directly applicable to English-language, user-facing AI assistant use in structured psychosocial-risk pre-assessment tasks where the user is an occupational health and safety professional and the task concerns risk identification, organizational framing, prevention-oriented action, and verification boundaries. They should be transferred more cautiously to informal employee self-use, clinical or legal decision-making, highly sector-specific risk assessment, or unstructured AI use without fixed prompts and professional oversight.

The scenario structure also limits the robustness claims that can be made. Although the study generated 60 conversations and 240 task-level outputs, these observations originated from four locked psychosocial-risk scenarios rather than from 240 independent workplace situations. This design strengthened controlled cross-product comparison by holding the underlying cases constant, but it does not exhaust the diversity of psychosocial risk management contexts, sectors, occupations, organizational cultures, or hazard combinations. Future studies should expand the scenario library and test whether the observed support-profile pattern remains stable across broader and more heterogeneous case sets.

A related limitation is that the benchmark used a single fixed professional profile: an occupational health and safety specialist with 5 years of experience in a medium-sized enterprise. This supported standardization and prevented user-role variation from becoming a confound, but it limits transferability to other potential users, such as human resources professionals, line managers, worker representatives, consultants, inspectors, clinicians, or occupational health and safety professionals with different levels of authority and experience.

A further boundary concerns temporal validity, which is inherent to benchmarks of user-facing AI assistant products. The benchmark was conducted between 19 and 25 March using the visible product environments and runtime labels available at that time. The findings should therefore be read as a time-stamped visible-output benchmark, rather than as fixed claims about all later versions, interface changes, access conditions, or model updates. This does not weaken the benchmark logic, but defines the period and product environment to which the observed patterns apply. For organizational use, especially where AI assistants are used repeatedly in occupational-health-relevant support tasks, comparable evaluations should be periodically repeated, for example annually or sooner when major model, interface, access-mode, or product-label changes occur.

The English-only protocol should also be read as a scope condition rather than as evidence about multilingual performance. English was used to standardize the benchmark across products and to keep the visible-output comparison fixed. The findings therefore apply to the English-language benchmark setting tested here and should not be generalized automatically to multilingual or non-English workplace contexts without further evaluation.

A further limitation concerns the shape of the final three-band distribution. Only one conversation was retained in the Problematic Support band, whereas 23 fell into Mixed/Partially Usable Support. Under the bounded prompt setting used here, the benchmark therefore discriminated more strongly between structurally usable and mixed support than between mixed and fully Problematic Support. This does not invalidate the typology, but it suggests that future work should test whether the problematic band expands under harder scenarios, less bounded prompts, or different operational settings.

### Closing interpretation

Taken together, the benchmark indicates that visible AI assistant outputs in psychosocial risk management differ in patterned ways that matter for professional usability. The findings support reading benchmark variation as a domain-specific support-pattern problem rather than as a generic comparison of consumer-facing assistant performance.

## Conclusion

This study introduced and applied an occupational health psychology and occupational health and safety-grounded benchmark for evaluating visible artificial intelligence assistant outputs in psychosocial risk management. Under the final consensus structure, the benchmark yielded a stable three-band support pattern, with 36 conversations classified as Structurally Usable Support, 23 as Mixed/Partially Usable Support, and 1 as Problematic Support.

The findings show that domain-specific AI evaluation in psychosocial risk management can be made transparent and reviewer-auditable through locked scenarios, fixed task sequences, visible-output archiving, task-aligned scoring, blind second-rating, and focused adjudication. The study’s contribution is therefore a structured evaluation logic for a high-stakes occupational use context, not a general product ranking or formal validation of a psychometric measurement instrument.

As workplace AI assistants become more embedded in organizational support tasks, their evaluation should remain domain-specific, transparent, time-stamped, and professionally bounded. The benchmark, governance guide, and reporting guide presented here offer a practical basis for comparing visible-output support patterns while keeping AI use subordinate to professional judgment, organizational verification, and responsible occupational health and safety practice.

## Data Availability

The benchmark protocol, locked scenarios, task prompts, rubric anchors, and retained reliability note are provided in Supplementary File 1. Additional archived benchmark materials supporting the findings are available from the corresponding author upon reasonable request.
