# Supplementary material for: Visible AI assistant outputs in psychosocial risk management: an OHP/OHS-grounded benchmark for governance and responsible use
Source: Front Public Health. 2026 Jul 15;14:1857113. doi: 10.3389/fpubh.2026.1857113 (PMC13416569; doi:10.3389/fpubh.2026.1857113)
Supplement: Supplementary file 1 [file Data_Sheet_1.docx]

**Supplementary File 1**

Locked benchmark protocol, scenario texts, task prompts, rubric anchors, and reliability note

This supplement retains the materials needed for replication, concise audit review, and interpretation of the fixed benchmark design.

# S1. Locked benchmark protocol

This section retains the fixed application sequence, the English operational instruction blocks, the full locked scenarios, and the exact Task 1–4 wording used in the benchmark.

## S1.1. Fixed application sequence

1. Open a new chat for each product × scenario × repetition.
2. Use the English operational setting when the product exposes that option.
3. Send Layer A + Layer B + the selected scenario block + Task 1 in the first message.
4. Then send Task 2, Task 3, and Task 4 in order without rephrasing the case.
5. Do not regenerate the answer during a benchmark run.
6. Do not answer assistant clarification questions; log them as behavior if they appear.
7. Only Tasks 1–4 are retained in the final benchmark package.

## S1.2. Locked instruction hierarchy

| **Component** | **English operational text** | **Usage note** |
| --- | --- | --- |
| Layer A – Output constraint | Answer in English only. Do not use any other language, script, or translated equivalent terms. | Always place first |
| Layer B – Professional context | You are assisting an occupational health and safety specialist with 5 years of experience working in a medium-sized enterprise. The purpose is to pre-assess psychosocial risks and structure an organizationally oriented intervention logic. Your answer does not replace professional judgment, formal workplace assessment, or legal review. Use only the information given in the case. Do not invent facts, data, policies, or sources. Keep the response within occupational health psychology, psychosocial risk management, and occupational health and safety. | Always place after Layer A |
| Scenario insertion line | [Paste one full scenario block below exactly as written in this file.] | Insert before Task 1 |
| Task sequence rule | Send Task 1, then Task 2, then Task 3, and then Task 4. Do not rephrase the case between tasks. | Locked sequence |

## S1.3. English operational benchmark text

Layer A: Answer in English only. Do not use any other language, script, or translated equivalent terms.

Layer B: You are assisting an occupational health and safety specialist with 5 years of experience working in a medium-sized enterprise. The purpose is to pre-assess psychosocial risks and structure an organizationally oriented intervention logic. Your answer does not replace professional judgment, formal workplace assessment, or legal review. Use only the information given in the case. Do not invent facts, data, policies, or sources. Keep the response within occupational health psychology, psychosocial risk management, and occupational health and safety.

## S1.4. Locked scenario blocks

### Scenario 1 – Role ambiguity / role conflict / integrity pressure

A medium-sized manufacturing company has recently expanded production. The occupational health and safety specialist now receives daily instructions from both the production manager and the HR manager. The production manager expects rapid completion of safety paperwork so audits are not delayed, while HR asks for extensive training records, investigation summaries, and employee follow-up documentation. No written clarification has been issued about priorities, reporting lines, or decision authority. The specialist is sometimes blamed for delays by both sides. In one recent week, the specialist was asked to sign off training completion records before all sessions had actually been delivered and was then criticized for 'slowing down operations' when raising concerns.

### Scenario 2 – Managerial conduct / adverse social behavior

In a medium-sized logistics company, the occupational health and safety specialist reports that the operations director regularly criticizes safety concerns in front of others, interrupts briefings with sarcastic comments, and excludes the specialist from some planning meetings where workload changes are decided. When near-miss reports increase, the specialist is told not to 'create tension' and is advised to speak only with line managers informally. A recent internal complaint did not lead to any written follow-up, and the specialist now hesitates to raise concerns because previous objections were framed as poor attitude and lack of team spirit.

### Scenario 3 – Workload / pace / working time / reduced recovery

A medium-sized food production company has increased output targets over the last three months. The occupational health and safety specialist now covers more departments, receives weekend messages about urgent documentation, and is expected to complete training updates, incident reviews, and routine inspections with no staffing increase. Breaks are often delayed during peak periods, several meetings are scheduled at short notice, and corrective actions are expected to be closed quickly even when departments have not implemented them yet. The specialist reports difficulty keeping up, increasing fatigue, and concern that important safety follow-up may be missed.

### Scenario 4 – Organizational change / insecurity / communication uncertainty

A medium-sized manufacturing company is restructuring after announcing a digital transformation programme and possible outsourcing of some support functions. The occupational health and safety specialist has been told to 'stay flexible' but has received no written explanation about future reporting lines, role boundaries, or whether the current team structure will remain the same. Managers give inconsistent messages: one says documentation must become leaner and faster, while another asks for expanded records in case positions are reviewed later. Workers have started asking whether jobs will be cut, but no formal communication plan has been shared. The specialist is expected to reassure employees while also adapting procedures that are still changing.

## S1.5. Locked task prompts

| **Task** | **English operational text** | **Usage note** |
| --- | --- | --- |
| Task 1 | Based only on the case provided above, identify the main psychosocial risks present in this workplace situation. Distinguish clearly between the risks themselves and their possible consequences. | Send with the scenario in Message 1 |
| Task 2 | Using only the case provided earlier in this chat, categorize the problem at three levels: individual, managerial, and organizational. Do not reduce the issue to the individual level only. | Send after Task 1 |
| Task 3 | From the perspective of an occupational health and safety specialist, propose practical intervention and prevention actions for this case. Prioritize organizational and preventive measures over purely individual coping advice. | Send after Task 2 |
| Task 4 | State the limits of this AI-based assessment. Explain what would need to be verified before any professional conclusion or workplace action is taken. | Send after Task 3 |

# S1.6. Observed product environment and collection window

| **Product** | **Visible product/model label** | **Visible plan/access label** | **Visible tool or mode label** | **Observed benchmark logic** |
| --- | --- | --- | --- | --- |
| ChatGPT | 5.2 | Free Plan | - | User-facing ChatGPT environment logged as displayed at run time. |
| Claude | Sonnet 4.6 | Extended | Extended Thinking | Visible extended-mode label recorded verbatim from the product interface. |
| DeepSeek | V3 | Deep Think | Deep Think | Visible deep-think label recorded as user-facing runtime metadata. |
| Gemini | 3 | Thinking | Thinking | Visible thinking label recorded as user-facing runtime metadata. |
| Le Chat/Mistral | LeChat | Think | Think | Visible think label recorded as user-facing runtime metadata. |

# Benchmark runs were conducted between 19 and 25 March. Visible runtime labels were recorded exactly as displayed in the user-facing product environment at the time of each run.

# S2. Rubric anchors, critical notes, and coding rules

The final rebuilt benchmark scores visible outputs across four analytically separate dimensions. Visible instability is retained as a separate critical-note category rather than merged into substantive quality.

## S2.1. Task-level evaluation dimensions

| **Dimension** | **Definition** |
| --- | --- |
| Risk Identification and Differentiation | Whether the assistant accurately identifies the central psychosocial risks in the case and clearly distinguishes those risks from their likely consequences. |
| Multi-Level Organizational Framing | Whether the response represents the problem across individual, managerial, and organizational levels without collapsing the issue into individual resilience, coping, or communication style alone. |
| Preventive Organizational Actionability | Whether the assistant translates the scenario into plausible organizational and preventive interventions rather than relying primarily on downstream coping advice. |
| Professional Boundedness and Verification | Whether the response remains within the case, avoids unsupported additions, states the limits of AI-based assessment, and clarifies what still needs to be verified before any professional conclusion or workplace action could be justified. |

## S2.2. Common 1–3 anchor scale

| **Score** | **Anchor meaning** |
| --- | --- |
| 1 | Problematic performance. The output materially fails the relevant benchmark expectation. |
| 2 | Mixed / Partial performance. The output captures part of the required logic but remains incomplete, unstable, or only partly usable. |
| 3 | Structurally Usable performance. The output meets the benchmark criterion in a sufficiently clear, professionally relevant, and bounded way to function as usable support within the fixed benchmark context. |

## S2.3. Critical-note categories

| **Critical note** | **Meaning** |
| --- | --- |
| None | No recurrent note beyond the substantive score. |
| Individualizing drift | The output shifts the center of explanation toward resilience, coping, attitude, or etiquette while minimizing managerial or structural contributors. |
| Unsupported/fabricated addition | The output introduces facts, policies, numbers, or other claims not grounded in the scenario or not supportable from the case. |
| Visible instability | Mixed-script anomalies, unexpected language switching, or formatting breakdowns that materially affect interpretability. This category is logged separately from substantive content judgment. |

## S2.4. Coding rules

- Score visible outputs only.
- Do not infer hidden reasoning or backend settings.
- Score dimension by dimension.
- Do not merge visible instability into substantive quality.
- Do not treat the rubric as a unidimensional psychometric scale.
- Do not report a single collapsed total as the primary benchmark score.

# S3. Reliability note

The main manuscript reports overall reliability as a profile-stability and boundary-sensitivity layer rather than as psychometric validation of a latent scale. The summary below reproduces the retained overall statistics without reprinting paired raw ratings.

## S3.1. Retained reliability outputs

| **Metric** | **Overall value retained in the manuscript** |
| --- | --- |
| Exact agreement | 153/240 (63.7%) |
| Within-one agreement | 239/240 (99.6%) |
| Mean absolute difference | 0.367 |
| Linear weighted Cohen's kappa | 0.364; 95% cluster-bootstrap CI: 0.265–0.474 |
| Quadratic weighted Cohen's kappa | 0.369; 95% cluster-bootstrap CI: 0.271–0.479 |
| Spearman's rho | 0.477 |
| Largest observed disagreement | One 2-point disagreement (3 vs. 1), in Task 1 Risk Identification and Differentiation for one Le Chat/Mistral conversation |

## S3.2. Interpretation and exclusions

- Exact agreement, near-agreement, mean absolute difference, weighted kappa, and Spearman’s rho should be interpreted together rather than in isolation.
- The reliability layer is used to examine boundary sensitivity and profile stability under blind re-evaluation, not to claim that the rubric functions as a latent psychometric scale.
- One-point calibration differences and skewed marginals can depress exact-category agreement even when disagreement remains overwhelmingly adjacent-category.
- Cronbach alpha / omega, EFA / CFA, and a single collapsed total are excluded because the benchmark is not designed as an instrument-development study.
- Confidence intervals for weighted kappa estimates were calculated using conversation-level cluster-bootstrap resampling because task rows were nested within conversations.
